# Detection of *sdh*B Gene Mutations in SDHI-Resistant Isolates of *Botrytis cinerea* Using High Resolution Melting (HRM) Analysis

**DOI:** 10.3389/fmicb.2016.01815

**Published:** 2016-11-15

**Authors:** Anastasios Samaras, Panagiotis Madesis, George S. Karaoglanidis

**Affiliations:** ^1^Faculty of Agriculture, Forestry and Natural Environment, Laboratory of Plant Pathology, Aristotelian University of ThessalonikiThessaloniki, Greece; ^2^Institute of Applied Biosciences, Centre for Research and Technology HellasThessaloniki, Greece

**Keywords:** boscalid, fungicide resistance, gray mold, strawberry, succinate dehydrogenase gene

## Abstract

*Botrytis cinerea*, is a high risk pathogen for fungicide resistance development. Pathogen’ resistance to SDHIs is associated with several mutations in *sdh* gene. The diversity of mutations and their differential effect on cross-resistance patterns among SDHIs and the fitness of resistant strains necessitate the availability of a tool for their rapid identification. This study was initiated to develop and validate a high-resolution melting (HRM) analysis for the identification of P225H/F/L//T, N230I, and H272L/R/Y mutations. Based on the sequence of *sdh*B subunit of resistant and sensitive isolates, a universal primer pair was designed. The specificity of the HRM analysis primers was verified to ensure against the cross-reaction with other fungal species and its sensitivity was evaluated using concentrations of known amounts of mutant’s DNA. The melting curve analysis generated nine distinct curve profiles, enabling the discrimination of all the four mutations located at codon 225, the N230I mutation, the three mutations located in codon 272, and the non-mutated isolates (isolates of wild-type sensitivity). Similar results were obtained when DNA was extracted directly from artificially inoculated strawberry fruit. The method was validated by monitoring the presence of *sdh*B mutations in samples of naturally infected strawberry fruits and stone fruit rootstock seedling plants showing damping-off symptoms. HRM analysis data were compared with a standard PIRA–PCR technique and an absolute agreement was observed suggesting that in both populations the H272R mutation was the predominant one, while H272Y, N230I, and P225H were detected in lower frequencies. The results of the study suggest that HRM analysis can be a useful tool for sensate, accurate, and rapid identification of several *sdh*B mutations in *B. cinerea* and it is expected to contribute in routine fungicide resistance monitoring or assessments of the effectiveness of anti-resistance strategies implemented in crops heavily treated with botryticides.

## Introduction

*Botrytis cinerea* Pers. ex Fr (teleomorph *Botryotinia fuckeliana* de BaryWhetz) the causal agent of gray mold disease, is an ubiquitous pathogen attacking more than 200 host plants. Within its host range are included some of the economically most important crops grown in field or in greenhouse, such as grapes, tomatoes, lettuce, strawberries, apples, pears, as well as several ornamental plants etc. Under favorable climatic conditions the fungus expands rapidly and may lead to devastating yield losses (Dean et al., 2012). An integration of cultural control methods may be used to combat the disease, such as environmental manipulation and sanitation, however, besides these preventive measures, chemical control remains the main tool for the successful disease management under conditions favorable for the development of the pathogen.

Fungicides belonging in several chemical groups are registered for use against the pathogen in most places of the world (Hahn, 2014). Among them, SDHIs represent the latest addition in the growers‘ quiver against the pathogen. They are currently the fastest growing fungicide group, including, in total 18 compounds (FRAC, 2016). Boscalid was the first SDHI molecule used against the pathogen in 2007, while later on fluopyram, isopyrazam, and penthiopyrad were also introduced as botryticides (Stammler et al., 2008; Veloukas and Karaoglanidis, 2012; Fillinger and Walker, 2016). Molecules belonging in the SDHI group share a common mode of action, consisting in the inhibition of respiration by inhibiting complex II, also known as succinate dehydrogenase, in the respiratory chain that leads to the block of the cell energy production (Cecchini, 2003).

However, the polycyclic nature of the disease, the high genetic variability, the abundant sporulation, the short generation time of the pathogen and the necessity for repetitive spray applications under favorable environmental conditions for disease development are factors contributing to a high risk for resistance development to site-specific fungicides used for its control. Indeed, in the past *B. cinerea* populations originating from several hosts such as grapes, strawberries, or tomatoes, throughout the world, have developed resistance to all the groups of site-specific fungicides used as botryticides that was associated with either target site modifications or overexpression of drug-eﬄux transporters (Leroux, 2007; Hahn, 2014; Fillinger and Walker, 2016).

SDHIs are considered to be of medium to high risk for resistance development. Resistance to boscalid, the first SDHI molecule introduced as a botryticide, emerged soon after the onset of its use. Currently, resistance to SDHIs has been reported in *B. cinerea* populations originating from table grapes, strawberry, tomato, lettuce, kiwi fruit, and apple fruit in several European and North or South America countries (Bardas et al., 2010; Kim and Xiao, 2010; Leroux et al., 2010; Leroch et al., 2011; Veloukas et al., 2011; Fernandez-Ortuno et al., 2012; Chatzidimopoulos et al., 2013; Amiri et al., 2014; De Miccolis Angelini et al., 2014b; Hahn, 2014; Konstantinou et al., 2015). Sequence analysis of the four *sdh* subunits in SDHI-resistant strains revealed that resistance was associated with nine target site mutations located in the *sdh*B subunit and one mutation in the *sdh*D subunit. In the *sdh*B subunit mutations associated with resistance to SDHIs have been detected at codons 225, 230, and 272 (Leroux et al., 2010; Veloukas et al., 2011; De Miccolis Angelini et al., 2014a; Esterio et al., 2015). At codon 225 the proline can be substituted by phenylalanine (P225F), leucine (P225L), threonine (P225T), or histidine (P225H). At codon 230 a single mutation has so far been detected, the substitution of asparagine by isoleucine (N230I), while at codon 272 the histidine may be replaced by arginine (H272R), tyrosine (H272Y), leucine (H272L), or valine (H272V). In the *sdh*D subunit the replacement of histidine by arginine at codon 132 (H132R) has been found to be associated with resistance to SDHIs in *B. cinerea* (Leroux et al., 2010).

For the detection of these mutations several techniques have so far, been developed. These techniques include direct sequencing of the four *sdh* subunits of *B. cinerea* (Leroux et al., 2010), allele-specific PCR enabling specific detection of two of the most common mutations associated with resistance to SDHIs in *B. cinerea*, H272R/Y (Yin et al., 2011), and a PIRA–PCR technique developed for the detection of H272L/R/Y, N230I, and P225F mutations (Veloukas et al., 2011). The above mentioned techniques require the fungal isolation, while multiple reactions are necessary to identify the mutations associated with the resistance. In addition to the above mentioned techniques, a newer method, that of pyrosequencing, was developed offering the advantage of quantification of several *sdh*B mutations in *B. cinerea* populations (Gobeil-Richard et al., 2015)

High Resolution Melting Analysis (HRM) is a novel and powerful molecular technique that has been introduced in 2002 and currently represents one of the simplest methods for genotyping, mutation scanning, and sequence matching (Reed et al., 2007; Taylor, 2009; Mondini et al., 2011). It is a PCR-based closed tube method that identifies variations in DNA sequence by measuring changes in fluorescence level of the melting DNA amplicon (Mondini et al., 2012; Sakaridis et al., 2014). Prior to HRM analysis, the region of interest is amplified using PCR in the presence of a dsDNA binding dye, while after PCR, melting curves are generated by monitoring the fluorescence of the binding dye in the presence of dsDNA. The whole process is monitored by a specific HRM instrument’s software resulting in a characteristic melting profile of the amplicon (Reed et al., 2007; Ganopoulos et al., 2012). Changes in the shape of the melting curves are due to differences in the sequence of the PCR amplicon (length, GC content, and actual sequence), (Mondini et al., 2015). HRM analysis has several advantages over other genotyping and scanning methods and due to these advantages has already started to be applied in several research aspects of plant pathology that include pathogen diagnosis and identification (Bester et al., 2012; Gori et al., 2012; Wong et al., 2013; Papavasileiou et al., 2016) or detection of mutations associated with resistance of plant pathogens to fungicides (Curvers et al., 2015).

Recently, an HRM assay has been developed and used for the detection of resistance to SDHIs in *B. cinerea* (Chatzidimopoulos et al., 2014). However, the developed assay was restricted only, in the detection of H272R/Y mutations. Therefore, a study was initiated aiming to investigate whether an expansion of the detection spectrum is possible, by developing a single HRM assay for the simultaneous detection and identification of eight mutations located in the *sdh*B subunit of *B. cinerea* that are associated with resistance to SDHIs. The accuracy of the developed assay was evaluated by identifying the *sdh*B mutations in *B. cinerea* samples obtained from strawberry fruit and stone fruit rootstock seedlings attacked by the pathogen.

## Materials and Methods

### Fungal Isolates

For the development of the method,14 *B. cinerea* isolates that were resistant to SDHIs and possessed eight distinct *sdh*B mutations (P225H/F/L/T, N230I, and H272L/R/Y) and two isolates that were sensitive to SDHIs (isolates of wild-type sensitivity) were included in the study. Most of the isolates had been collected from Greek strawberry fields or stone fruit rootstocks cultivated in the greenhouse, for the requirements of monitoring studies aiming to identify *sdh*B mutations associated with resistance to SDHIs (Veloukas et al., 2011; Konstantinou et al., 2014, 2015). The *sdh*B mutations had been identified using a PIRA–PCR technique described previously (Veloukas et al., 2011) or direct sequencing of the *sdh*B gene (Leroux et al., 2010). The isolates possessing the P225L and P225T mutations were originating from Germany. All the isolates were identified as *B. cinerea* using a duplex PCR assay developed by Plesken et al. (2015). A complete list of the isolates used in the study and their origin is provided in **Table 1**.

**Table 1:** List of *Botrytis cinerea* isolates used for the development of the assay.

Isolate code	*sdh*B mutation^a^	Year of isolation	Origin
BcSV10	Wild-type	2014	strawberry
Bc33	Wild-type	2010	strawberry
BcSv14	H272R	2014	strawberry
BcTPR48	H272R	2012	tomato
BcSv44	H272Y	2014	strawberry
Bc30	H272Y	2011	strawberry
BcE114	H272L	2011	strawberry
Bc278	H272L	2009	strawberry
BcE98	N230I	2011	strawberry
BcTKp12	N230I	2011	tomato
BcS22	P225F	2010	strawberry
BcS24	P225F	2010	strawberry
BcM100	P225H	2013	Cherry rootstock
BcM182	P225H	2013	Cherry rootstock
BcB1	P225L^b^	–	grape
BcB2	P225T^b^	–	Laboratory
			induced mutation

*^a^*sdh*B mutations were identified using a PIRA–PCR technique (Veloukas et al., 2011) and confirmed by direct sequencing of *sdh*B using the primer pair IpBcBeg-IpBcend (Leroux et al., 2010). ^b^Isolates possessing the P225L and P225T mutations were kindly offered to us by Dr. Gerd Stammler (BASF, Limburgerhof, Germany).*

### Sequencing of the Isolates

**FIGURE 1:**
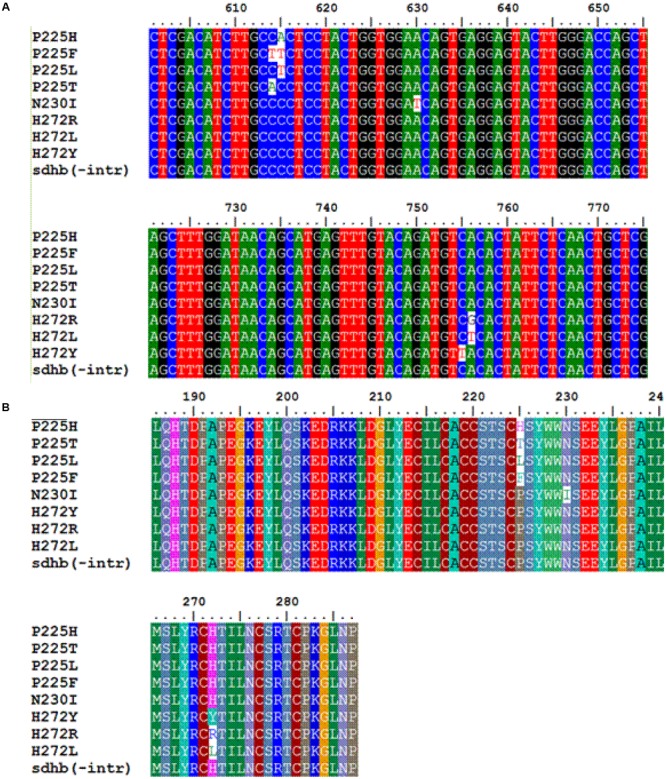
**Multiple sequence alignment of nucleotides (A)** and amino acids **(B)** of the *sdh*B gene in SDHIs resistant isolates of *Botrytis cinerea* with eight different mutations in subunit B and one sensitive isolate.

To confirm the identification obtained using the PIRA–PCR technique and to determine the specific nucleotide polymorphisms associated with each amino acid change the entire *sdhB* sequence was amplified using the primer pair IpBcBeg (5′-CCACTCCTCCATAATGGCTGCTCTCCGC-3′) and IpBcEnd2 (5′-CTCATCAAGCCCCCTCATTGATATC-3′) designed by Leroux et al. (2010). Amplification conditions were performed as those described by Leroux et al. (2010). PCR products were separated by electrophoresis in 1% agarose gel in 1x TAE buffer and visualized after ethidium bromide staining under UV light. PCR products were purified using the Qiaquick PCR Purification Kit (Qiagen GmbH, Hilden, Germany). The purified products were subjected to sequencing and sequences were aligned using the computer software package Mega 5.05. The nucleotide polymorphisms associated with each mutation are shown in **Figure 1**.

### Primer Design and HRM Conditions

Based on the sequence of the *sdh*B subunit of a reference strain (B05.10) a primer set was designed (sdhB-Fw: GAGGATCGTAAGAAGCTTGATGG and sdhB-Rev: TGTCTTAGTCTCCGCAATTGCC), expected to amplify a 249 bp product. The primer pair was designed using the software program Vector NTI (ThermoFisher Scientific).

PCR amplification, DNA melting and end point fluorescence level acquiring PCR amplifications were performed in a 20 ml volume containing 20 ng of genomic DNA, 4 μM of SdhB-Fw and SdhB-Rev primers, 0.2 mM of each dNTP, 2.5 mM MgCl_2_, 1.5 mM SYTO 9 Green fluorescent nucleic acid stain (Life Technologies Corp.), and 1 U Kapa Taq DNA polymerase (Kapa Biosystems). The PCR amplifications were performed in a Rotor-Gene 6000 real-time 5P HRM PCR thermocycler (Corbett Research) using the following thermal parameters: an initial preheat for 10 min at 95°C, followed by 35 cycles at 95°C for 20 s, 55°C for 20 s, 72°C for 15 s, and terminated with a final extension at 72°C for 10 min. After the PCR amplification, HRM was performed from 80 to 88°C rising by 0.1°C in each step with a 2 s hold time for each step with 90 s of pre-melt conditioning on first step. The HRM data were analyzed with the Rotor-Gene Q series software version 2.3.1 (Qiagen, Milan, Italy). The melting curves were normalized in order to limit the temperature range between the pre- and post-melt regions and eliminate the fluorescence variance. All the reactions were repeated in triplicate.

### Assay Specificity

The specificity of the designed primers was evaluated against several fungal species that can be found on strawberry fruit either as pathogens or contaminants on rotten fruit tissues, such as *Alternaria alternata, Rhizopus stolonifer*, and *Aspergillus niger*. The pathogens used were belonging in the fungal culture collection of the Laboratory of Plant Pathology. One isolate of each pathogen was used in the assays. PCR amplifications and the HRM assay were performed using in the same reaction DNA of the above mentioned pathogens along with DNA of *B. cinerea* isolate BcSV10. Amplification and HRM assay conditions were as described previously. Comparison of melting peaks and similarity tests were conducted using Rotor-Gene Q series software version 2.3.1.

### Assay Sensitivity

To determine the detection limits of the developed HRM assay, an analysis was performed using known DNA concentrations of the H272R mutant BcTPR48 and the isolate of wild-type sensitivityBcSV10. Concentrations of 0.001, 0.01, 0.1, 1, 10 ng per reaction tube were used. The PCR amplification and HRM analysis conditions were the same as described above.

### Application of HRM on DNA Samples Extracted from Fruit Tissues

The 16 isolates listed in **Table 1** that had been used to develop the HRM method using DNA samples from pure fungal cultures were also used to artificially inoculate strawberry fruit (cv. Camarosa). Artificial inoculations and incubation conditions were as described by Veloukas and Karaoglanidis (2012). After the end of the incubation period 100 mg of rotten fruit tissue were removed and used for DNA extraction. The extraction was conducted using Nucleospin Plant II kit (Marchery–Nagel GmbH & Co.), following the manufacturer’s protocol.

The PCR amplification and HRM analysis conditions were as described previously. To compare melting curves, DNA samples of the *sdh*B mutants that had been extracted from rotten tissue were used in the same reactions along with DNA samples extracted from pure cultures of the mutants. Comparisons of the melting curves were conducted using the Rotor-Gene Q series software version 2.3.1. All the reactions were repeated in triplicate.

### Validation of Assay Accuracy in Naturally Infected Strawberry Fruit and Stone Fruit Seedling Samples

To test the reliability of the developed assay in identifying *sdh*B mutations in field samples, an extensive sampling was conducted during the spring of 2015 in strawberry fields located in the region of Pieria, northern Greece and in greenhouses located in the region of Arta, western Greece, where seedlings of stone fruit rootstocks were growing. Strawberry fruit showing gray mold symptoms were collected, at harvest period, from three different strawberry fields. Similarly, from the greenhouses seedlings showing symptoms of damping – off disease due to *B. cinerea* infection were collected. The strawberry fruit or the stone fruit rootstock seedling plants were transferred to the laboratory in individual polyethylene bags to prevent cross contamination. From each sample a *B. cinerea* single-spore isolate was obtained using a procedure described previously (Myresiotis et al., 2007). In total, 50 and 134 single-spore isolates were collected from strawberry fruit and the rootstock seedlings, respectively. The isolates were characterized as boscalid-resistant or -sensitive using the discriminatory concentration of 2 μg ml^-1^ boscalid, using a procedure previously described (Konstantinou et al., 2015).

From the same fruit or seedling plants used for pathogen isolation, DNA was directly extracted from the rotten tissues as described previously and *sdh*B mutations in the samples were identified following the HRM assay protocol described above. The results of identification using the HRM assay were compared with identification of *sdh*B mutations in the respective samples using a standard PIRA–PCR technique developed by Veloukas et al. (2011). For those samples that identification of *sdh*B mutations was not possible with the PIRA–PCR, direct sequencing of the entire *sdh*B gene was conducted.

## Results

### HRM Analysis for the Identification of Mutations in *sdh*B Gene

**FIGURE 2:**
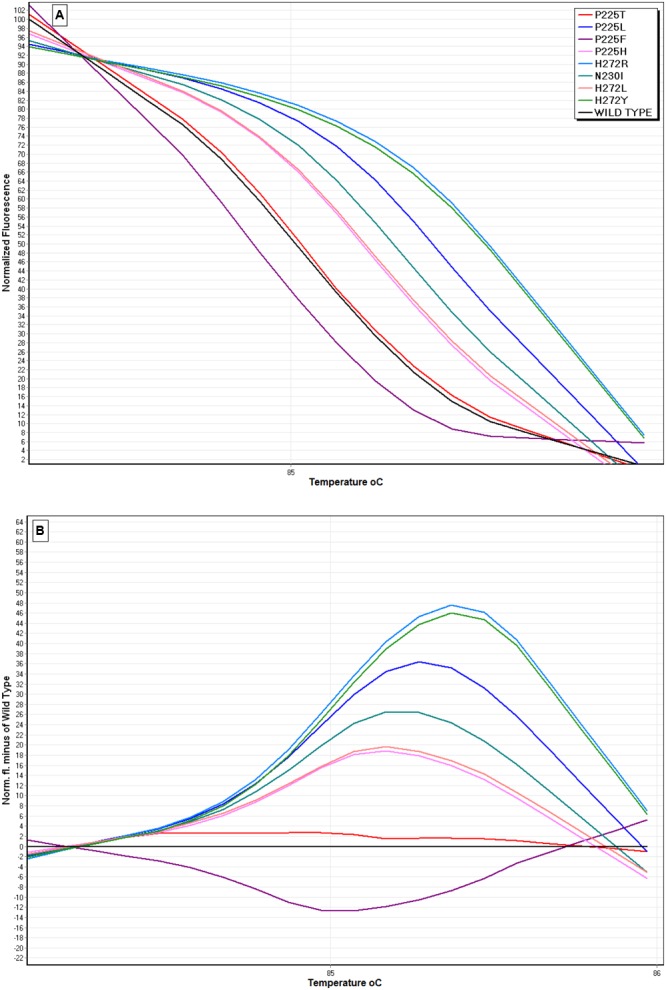
**High resolution melting (HRM) analysis using the primers SDHB-Fw and SDHB-Rev for the discrimination of SDHI-resistant *B. cinerea* isolates possessing the *sdh*B mutations P225H/T/F/L, H272R/Y/L, and N230I. (A)** Normalized HRM curves of isolates with different mutations, **(B)** Difference curves of HRM using a sensitive isolate as the reference baseline curve.

The primer pair SDHB-Fw and SDHB-Rev amplified a 249 bp fragment of the *sdh*B gene in all the nine *B. cinerea* genotypes included in the study. The nine different genotypes were distinguished by a shift in melting temperature axis providing a different melting profile (**Figure 2**). The HRM analysis generated nine distinct curves, one for each genotype. The shape and profile of normalized curves of each isolate were unique, allowing the discrimination of the *sdh*B mutations (**Figure 2A**). In addition, the difference graph generated using the wild-type genotype’ curve as the baseline, revealed part of the curve sitting outside the 80% confidence interval (CI) curve, indicating that the several mutants included in the study were, indeed, different (**Figure 2B**).

### Assay Specificity and Sensitivity

**FIGURE 3:**
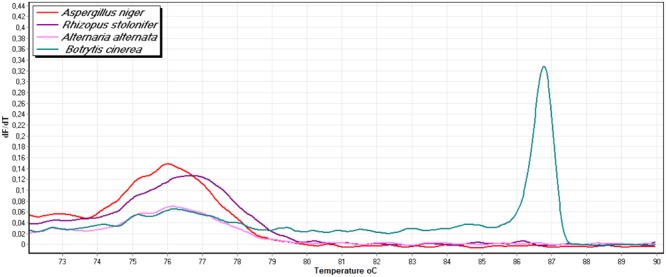
**Melting profile of four fungal pathogens, using the specific primers SDHB-Fw and SDHB-Rev with genomic DNA of *B. cinerea, Aspergillus niger, Rhizopus stolonifer*, and *Alternaria alternata***.

The primer pair SDHB-Fw and SDHB-Rev was designed based on the sequence of the *sdh*B gene of *B. cinerea*. The specificity assay was conducted against pathogens or contaminants that may grow on strawberry fruit such as *A. niger, A. alternata*, and *R. stolonifer.* The results showed that the designed primer pair was not able to amplify the 249 bp product in any of these fungal organisms but *B. cinerea* (**Figure 3**).

**FIGURE 4:**
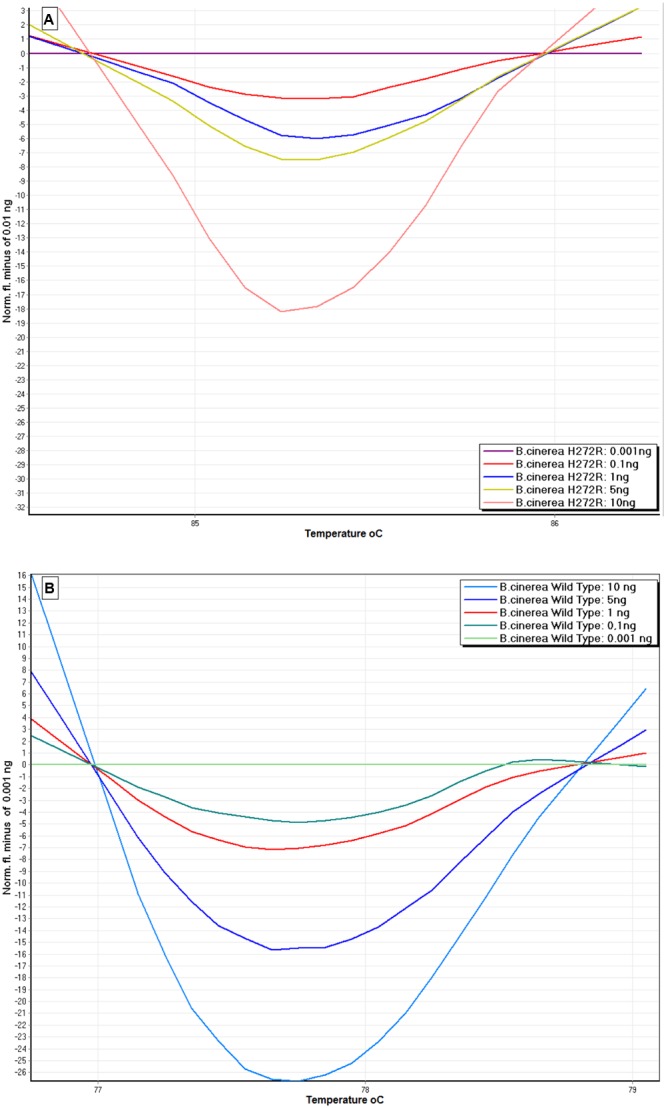
**Sensitivity of HRM assay to detect DNA of a *B. cinerea* isolate possessing the H272R mutation (A)** and a wild-type isolate **(B)**. Difference curves were generated using 0.1–10 ng DNA per reaction tube. 0.001 ng of genomic DNA from *B. cinerea* was used as reference baseline curve.

The assay was highly sensitive since the *SDHB-Fw and SDHB-Rev* specific primers successfully detected down to 0.01 ng of genomic DNA, as shown in the difference graph generated using 0.001 ng of BcTPR48 H272R mutant (**Figure 4A**) or of the wild-type isolate BcSV10 (**Figure 4B**), as the reference baseline curve.

### HRM Analysis for the Detection of Mutations in *sdh* Gene of *B. cinerea, In planta*

**FIGURE 5:**
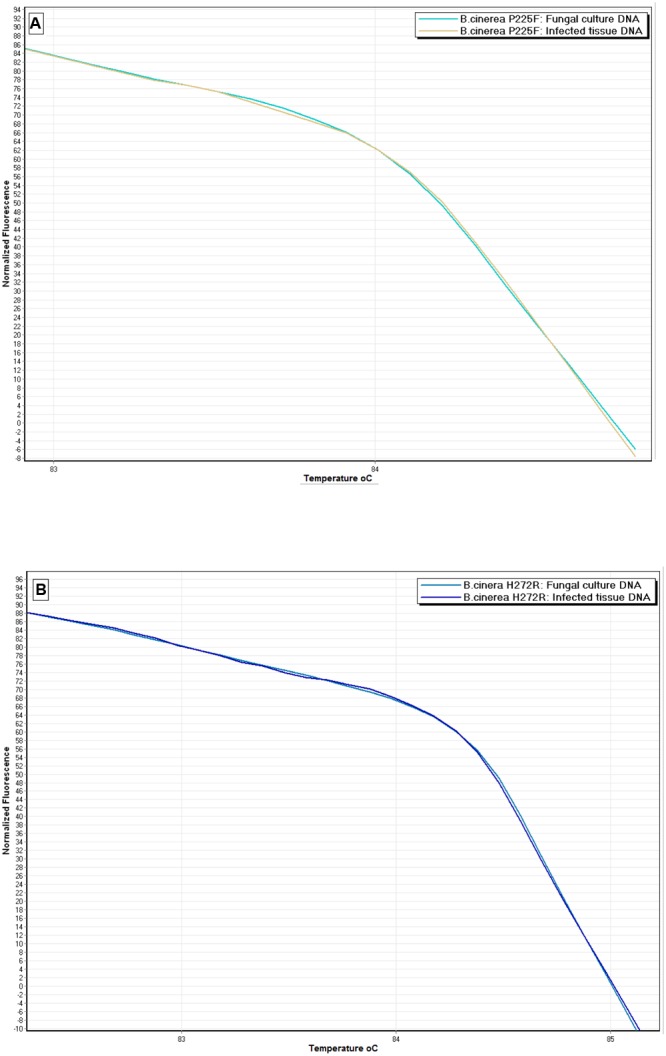
**Normalized HRM analysis curves of two *B. cinerea* isolates possessing P225F (A)** and H272R **(B)** mutations, generated from genomic DNA extracted from a fungal culture and from artificially inoculated strawberry fruit tissue.

When the developed protocol of HRM analysis was applied using DNA extracted directly from artificially inoculated strawberry fruits as a template, it was also shown to be efficient in discriminating the nine *B. cinerea* genotypes tested. As in the case of DNA extracted from pure cultures, nine distinct curves were generated corresponding to each of the nine genotypes tested. HRM analysis curves which were generated using DNA extracted from the strawberry tissues exhibited high similarity (>90% CI) to the HRM analysis curves generated using DNA from the pure fungal cultures. Representative HRM analysis curves of DNA originating from pure fungal culture and infected strawberry tissue are provided, as an example, for two out of nine genotypes tested (P225F and H272R) in **Figure 5**.

### Validation of Assay Accuracy in Naturally Infected Strawberry Fruit and Stone Fruit Seedling Samples

**FIGURE 6:**
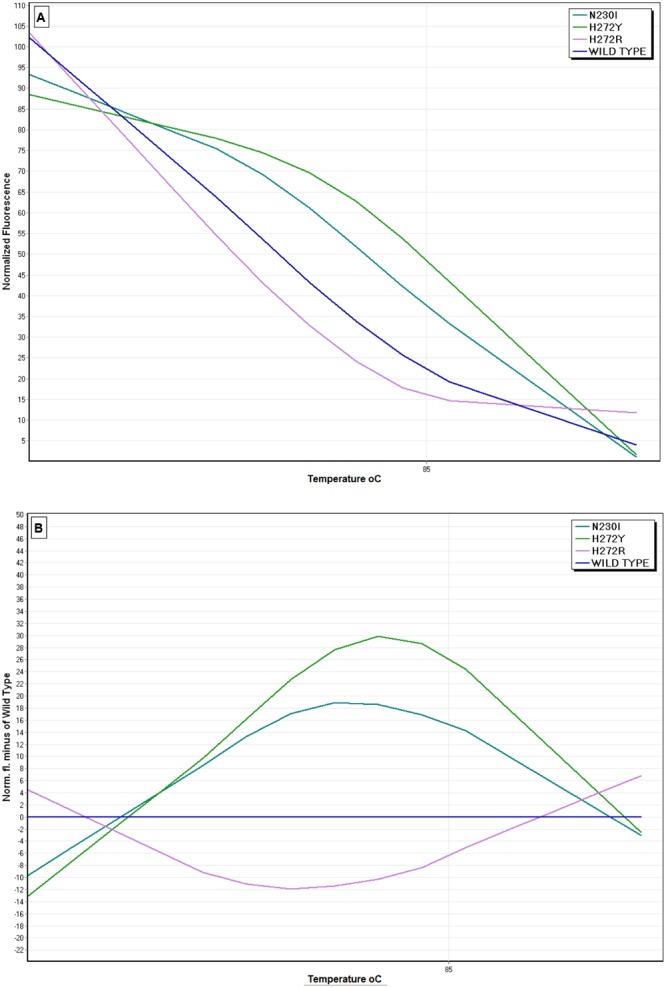
**Representative HRM analysis curves of four *B. cinerea* genotypes generated from unknown field samples. (A)** Normalized HRM curves of DNA samples with H27R/Y and N230I mutations and one non-mutated DNA sample (wild-type), **(B)** Difference graph of HRM curves using the non-mutated (wild-type) sample as the reference baseline curve.

Identification of *sdh*B mutations in *B. cinerea* samples from decayed strawberry fruit or stone fruit rootstock seedling plants showing damping-off symptoms showed that there was a perfect agreement among the novel HRM method and the standard PIRA–PCR method. Resistance detection using a standard bioassay in the 50 isolates obtained from strawberry fruit showed that 20 of them were sensitive to boscalid and the remaining 30 were resistant (**Table 2**). Similarly, in the 134 isolates obtained from the stone fruit rootstock seedlings, was found that 34 of them were sensitive and the remaining 100 resistant to boscalid (**Table 2**). The HRM analysis confirmed the presence of 20 and 34 samples without any mutation in *sdh*B subunit, in the *B. cinerea* DNA samples extracted from strawberry fruit and stone fruit rootstock plants, respectively (**Table 2**). Furthermore, HRM analysis showed that within the DNA samples from strawberry fruit tested there were 18, 11, and 1 samples harboring the H72R, H272Y, and N230I mutations, respectively (**Table 2**). All 30 mutated samples were typed to one of the three above mentioned genotypes with a confidence threshold >90%. Furthermore, all the three genotypes harboring mutations were clearly discriminated from the non-mutated samples (samples of wild-type sensitivity), (**Figure 6**). Similarly, in the samples obtained from stone fruit rootstock seedlings HRM analysis showed the presence of 77, 21, and 2 samples possessing the H272R, N230I, and P225H mutations, respectively (**Table 2**). The identification results obtained by the application of the HRM analysis were in absolute agreement, for all the samples, with identification data obtained by the standard PIRA–PCR technique. Due to absence of available PIRA–PCR method for the identification of P225H mutation, the presence of the mutation was confirmed by direct sequencing of the *sdh*B subunit.

**Table 2:** Determination of resistance to boscalid and detection of *sdh*B mutations using high resolution melting (HRM) analysis, PIRA–PCR, and conventional bioassays in *B. cinerea* samples obtained from infected strawberry fruit and stone fruit rootstock seedling plants showing damping-off symptoms.

mutant	Host						

	**Strawberry (*n* = 50**^**b**^)			**Stone fruit rootstock seedling plants (*n* = 134**^**b**^)		
	**HRM**	**PIRA–PCR**^**a**^	**Conventional bioassays**^**c**^	**HRM**	**PIRA–PCR**	**Conventional bioassays**
Wild-type	20	nd^d^	20	34	nd	34
H272R	18	18	nd^e^	77	77	nd
H272Y	11	11	nd	0	0	nd
N230I	1	1	nd	21	21	nd
P225H	0	0	nd	2	2^f^	nd

*^a^The PIRA–PCR protocol developed by Veloukas et al. (2011) was used. ^b^Number of samples/isolates. ^c^Conventional bioassays were conducted on YBA medium using the discriminatory concentration of 2 μg ml^-1^boscalid (Konstantinou et al., 2015). ^d^not determined (nd) since the PIRA–PCR detects only specific *sdh*B mutations. ^e^not determined (nd) since conventional bioassays discriminates only isolates of wild-type sensitivity from isolates with resistance to boscalid. ^f^P255H was identified by direct sequencing of the *sdh*B gene using the primer pair IpBcBeg and IpBcEnd2, designed by Leroux et al. (2010).*

## Discussion

The current study was conducted aiming to develop a novel method for rapid identification of *sdh*B mutations in *B. cinerea* isolates with resistance to SDHIs. HRM was selected since it represents a method that is increasingly popular and widespread due to its accuracy, easy, fast, and cost effective application. It has recently been also successfully adopted in a series of research applications that include single base change genotyping, insertion and deletion genotyping sequence matching, methylation profiling, internal tandem duplication detection, and mutation screening (Radvanszky et al., 2015), while it is in its infancy for the detection of mutations associated with the resistance of fungal plant pathogens to fungicides.

Currently, four SDHI molecules (boscalid, penthiopyrad, isopyrazam, and fluopyram) have been registered for use as botryticides in different places of the world, while some additional active ingredients such as benzovindiflupyr and isofetamid, may be introduced into the agricultural practice in the near future (Fillinger and Walker, 2016). The availability of different molecules belonging in the same fungicide class along with the observed variability in *sdh*B mutations associated with resistance to SDHIs makes necessary the development of a rapid method enabling the simultaneous detection of the several *sdh*B mutations. This is particularly important taking into account that the several *sdh*B mutations confer differential levels of resistance to the different SDHI molecules (Veloukas et al., 2013; Amiri et al., 2014; De Miccolis Angelini et al., 2014a). For instance, H272Y mutation confers moderate levels of resistance to boscalid or low levels of resistance to isopyrazam but it is associated with hypersensitivity to fluopyram. Similarly, H272R mutation is associated with moderate levels of resistance to boscalid or isopyrazam but it does not affect the sensitivity of the mutants to fluopyram. In contrast, other mutations such as H272L or P225F/L are associated with moderate or high levels of resistance to all the molecules of the SDHI group that are registered as botryticides. Furthermore, recent findings suggest that the different *sdh*B mutations may have adverse effects on the fitness of the mutated strains (Laleve et al., 2014; Veloukas et al., 2014). Although there are some discrepancies among these two studies a common conclusion can be derived suggesting that the different *sdh*B mutations have different impacts on the fitness of the strains. Such differences may have significant implications for the implementation of the management of resistance to SDHIs and therefore, precise knowledge of the population composition in terms of specific mutations presence and their frequency within the population is necessary.

In the recent past several methods were developed for the detection and identification of *sdh*B mutations in *B. cinerea*. These methods were including amplification and direct sequencing of the target gene (Leroux et al., 2010), a PIRA–PCR technique for the detection of H272L/R/Y, P225F, and N230I mutations (Veloukas et al., 2011), an allele-specific PCR assay for the detection of H272R/Y mutations (Yin et al., 2011) or more recently a PCR–RFLP technique for the detection of the N230I mutation (Amiri et al., 2014) and a quantitative real-time allele-specific PCR for the detection of N230I, P225F/L, and H272R/Y/V (De Miccolis Angelini et al., 2014a). However, all these methods require, as a first step, the isolation of the pathogen while, with the exception of the direct gene sequencing, the remaining methods require multiple reactions for the identification of the *sdh*B mutations associated with resistance.

In the present paper the development of a HRM assay for the rapid and simultaneous detection of eight different *sdh*B mutations is described. The assay successfully recognized the eight mutants, as well as the non-mutated strains of wild-type sensitivity to SDHIs. The nine genotypes tested provided melting curves of the PCR products with distinct and consistent shape and profile, enabling their discrimination. The melting curve profile of a PCR product is affected by several parameters, among which the GC/AT ratio, the length and sequence of the PCR product are included (Ririe et al., 1997; Reed et al., 2007). The primer pair used in this study was designed based on the sequence of the *sdh*B subunit of *B. cinerea*, the subunit in which the most common mutations associated with resistance of this fungus to SDHIs occur (Leroux et al., 2010). The designed primer pair amplified a 249 bp region of the gene in which the three codons, 225, 230, and 272, with possible mutations were included. The size of PCR product was of 249 bp length. Successful application of HRM analysis for SNP scanning requires PCR products of relatively small length. In case of PCR products with a length longer than 400 bp the error rate increases, and the sensitivity of the analysis decreases, while, in contrast, the position of the mutation within the PCR product does not affect the scanning accuracy (Reed and Wittwer, 2004; Reed et al., 2007). Interestingly, the method was found to be highly sensitive enabling discrimination of multiple SNPs located in the same codon (i.e., four different SNPs at codon 225 and three different SNPs at codon 272). Even when the HRM curves might look similar the software of the instrument can identify even slight differences and express these as confidence values which are the numerical data showing the similarities and differences of the different genotypes used in the analysis. Confidence value above 80% is considered significant. A similar HRM assay developed aiming to discriminate SNPs located at codon 54 of the *Cyp51A* gene of *Aspergillus fumigatus* proved to be successful in discriminating the mutated strains from the strains of wild-type sensitivity to the azole fungicides but failed to discriminate the seven different mutants from each other (Tuohy et al., 2010).

The method was capable of discriminating the *sdh*B mutants, included in the study, using as template DNA isolated both from pure fungal cultures or infected fruit tissues. This offers an extra advantage for the test contributing to the rapid identification of *sdh*B mutations without the need for previous isolations of the pathogen. In addition to the rapidity of the assay it was shown that it was highly specific. One of the main problems faced with methods that use DNA directly extracted from the plant tissues is the possible presence of infections by multiple pathogens or contaminants on the tissue surface (Gori et al., 2012). The primer pair designed for the assay specifically discriminated the DNA of *B. cinerea* from that of other fungal species that may infect or may grow as saprophytes on strawberry fruit. Similarly, the simultaneous presence in the infected tissue of different *B. cinerea* strains cannot be excluded. However, taking into account that the assay is highly sensitive it is expected that different genotypes that may co-exist in the tissue sample are easily discriminated.

The developed assay was validated by applying that in unknown Botrytis samples originating from either infected strawberry fruit or infected seedlings of stone fruit rootstocks showing damping-off symptoms. The data obtained by the HRM analysis were compared with the data derived by the phenotyping of the isolates using a conventional bioassay technique as well as by the application of a standard PIRA–PCR technique developed by Veloukas et al. (2011) to identify P225F, N230I, and H272L/R/Y mutations. The comparison showed that the HRM assay can distinguish the isolates of wild-type sensitivity from the *sdh*B mutants that were found to be phenotypically resistant to boscalid, while in addition the assay enabled the detection of the *sdh*B mutations in the SDHI-resistant samples. In regards of *sdh*B mutation detection the HRM assay provided similar results with the standard PIRA–PCR technique or the sequencing of the entire gene.

Both crops that were monitored by the HRM assay are heavily treated with fungicides against *B. cinerea*. In Greece strawberries are cultivated under high height plastic tunnels and more than six spray fungicide applications are required for successful control of the disease. Similarly, stone fruit rootstock seedling plants are transferred after their generation by micro-propagation in the greenhouse for acclimatization and initial growth. Their need for constant high humidity creates a microenvironment extremely favorable for the development of the disease and for its control successive applications of botryticides are required. HRM analysis showed that, on both hosts, the substitution of histidine by arginine at codon 272 (H272R) was the most common mutation in the SDHI-resistant fraction of the fungal populations. This is in agreement with numerous previous monitoring studies from all over the world suggesting that H272R is currently the most common mutation associated with resistance to SDHIs in *B. cinerea* (Leroux et al., 2010; Veloukas et al., 2011; Fernandez-Ortuno et al., 2012; De Miccolis Angelini et al., 2014a). Other mutations such as H272Y or N230I were found in lower frequencies, while P225H was detected in a very low frequency in the population originating from stone fruit rootstock seedlings. P225H mutation is a rather rare mutation that, till now, has been reported only in *B. cinerea* originating from stone fruit rootstock seedlings in Greece (Konstantinou et al., 2014) and from table grapes in Chile (Esterio et al., 2015). In most of the monitoring studies published previously these mutations are rare or in lower frequencies compared to the frequency of H272R mutation. This is probably correlated with a fitness cost that has been confirmed recently both for isogenic and field strains of the pathogen possessing mutations such as the N230I, P225L/F, or the H272L (Laleve et al., 2014; Veloukas et al., 2014).

The HRM protocol developed in the current study has many advantages compared to other methods developed previously for the detection of *sdh*B mutations in *B. cinerea* (Veloukas et al., 2011; Yin et al., 2011; Amiri et al., 2014). The most important one is the fact that expands the detection spectrum to all the currently known *sdh*B mutations, conferring resistance to SDHIs in *B. cinerea*. In total, by this method eight different mutations can be detected in a single reaction. In addition, the developed protocol enables the detection of sequence variations within the PCR product without the need of a separation step achieved by gel electrophoresis that is required in older molecular methods. This separation often takes hours to perform and increase the risk for contamination. Furthermore, the developed assay allows the handling of several samples in a single reaction, while older methods necessitate the application of several reactions, often with the need for changing primers. In general, the HRM analysis is a single tube post PCR reaction which is fast, highly accurate, and cost effective since does not require gel analysis or sequencing of the PCR products or any other post PCR treatment.

However, the developed method has also some limitations. Among these the most important one is that there is a controversial need for reference controls. The simultaneous presence of both the reference and the unknown samples allows direct comparison of the melting curves, since it is possible that slight variations in the confidence levels may occur between runs (Robertson et al., 2009; Wong et al., 2013). However, this disadvantage might be overcome by obtaining a melting curve database to serve as standard (Madesis et al., 2014). Another disadvantage of the developed method is that it is a qualitative one and does not offer quantitative data for the frequency of each genetic substitution within a pathogen’ population. However, it has been proposed that HRM analysis can be combined with qPCR in case that quantitative data are required (Rouleau et al., 2009). Currently, the only available method for quantitative assessment of *sdh*B mutants in *B. cinerea* is a recently developed pyrosequencing-based method (Gobeil-Richard et al., 2015). A third disadvantage of the method that has to be taken into account, when it is applied in fungicide resistance monitoring programs, is the probability for the presence of natural polymorphisms in the amplified products that might change the melting profile of the amplicon leading to false results. Such polymorphisms are not necessarily associated with resistance to SDHIs (e.g., silent mutations). Actually, this probability is very high when genes with high frequency of natural polymorphisms are amplified, such as, for instance, the 3-ketoreductase (*erg*27) gene, the target site of hydroxyaniline fungicides (Grabke et al., 2013). Yet, in the case of *sdh*B mutants the probability is low since the gene is considered to be highly conserved and natural polymorphisms are very rare. In addition, as already stated the amplified part of the genome by the designed primer pair, is not excessively long and the risk for natural polymorphisms existence is further reduced.

The development of fungicide resistance in *B. cinerea* populations is one of the major limiting factors of successful control of the pathogen (Hahn, 2014). SDHIs, in particular, as being one of the latest pesticide additions used against the pathogen may play a crucial role in the implementation of anti-resistance strategies since they do not show cross-resistance relationships with fungicides of other chemical classes in which the pathogen has already developed resistance. But taking into account, that they are also fungicides of medium to high risk for resistance development, continuous monitoring are required for sensitivity measurements and detection of specific pathogen mutants with resistance to this fungicide class. In conclusion, the HRM method developed in the current study could easily be adopted as the method of choice for a rapid identification of *sdh*B mutations in *B. cinerea* using pathogen DNA either extracted from pathogen colonies or from infected plant tissues from laboratories that are equipped with the necessary instruments. Due to its low cost and the reduction in time required to obtain results it can be applied in programs aiming to monitor the presence of specific *sdh*B mutations in pathogens’ populations in crops that heavily treated with botryticides. Such knowledge is a prerequisite for the successful implementation of strategies aiming to prolong the use of SDHIs as botryticides.

## Author Contributions

GK and PM conceived and designed the experiments. AS performed all experiments, analyzed the data, and wrote part of the paper. GK supervised the study and wrote the paper. All authors read and approved the final manuscript.

## Conflict of Interest Statement

The authors declare that the research was conducted in the absence of any commercial or financial relationships that could be construed as a potential conflict of interest.
